# Evaluating Moisture Content in Immersion Vacuum-Cooled Sausages with Citrus Peel Extracts Using Hyperspectral Imaging

**DOI:** 10.3390/life14050647

**Published:** 2024-05-20

**Authors:** Chao-Hui Feng, Hirofumi Arai, Francisco J. Rodríguez-Pulido

**Affiliations:** 1 School of Regional Innovation and Social Design Engineering, Faculty of Engineering, Kitami Institute of Technology, 165 Koen-cho, Kitami 090-8507, Japan; araihrfm@mail.kitami-it.ac.jp; 2 RIKEN Centre for Advanced Photonics, RIKEN, 519-1399 Aramaki-Aoba, Aoba-ku, Sendai 980-0845, Japan; 3 Food Colour and Quality Laboratory, Facultad de Farmacia, Universidad de Sevilla, 41012 Sevilla, Spain; rpulido@us.es

**Keywords:** immersion vacuum cooling, modified casings, hyperspectral imaging, sausage, Kennard–Stone algorithm

## Abstract

The moisture content of immersion vacuum-cooled sausages with modified casings containing citrus fruit extracts under different storage conditions was studied using hyperspectral imaging (HSI) associated with chemometrics. Different pre-processing combinations were applied to improve the robustness of the model. The partial least squares regression model, employing the full reflectance spectrum with pre-treatment of the standard normal variate, showed calibration coefficients of determination (R_c_^2^) of 0.6160 and a root mean square error of calibration (RMSEC) of 2.8130%. For the first time, prediction maps developed via HSI visualized the distribution of moisture content in the immersion vacuum-cooled sausages with unique modified casings in response to fluctuating storage conditions. The prediction maps showed exact parts with high water content, which will help us to monitor and prevent mold growth. The combination of HSI with multivariate analysis not only quantifies changes in moisture content but also visually represents them in response to various casing treatments under different storage conditions, illustrating the significant potential for real-time inspection and early mold detection in sausages within the processed meat industry.

## 1. Introduction

Cooling is a prevalent technique employed to preserve the quality of agricultural and food products. Among various cooling techniques, immersion vacuum cooling (IVC) is distinguished as an innovative approach, showcasing a rapid cooling rate when compared to conventional methods such as air blast (AB) and immersion cooling (IC). Additionally, IVC exhibits significantly less cooling loss compared to vacuum cooling (VC) [1,2,3,4,5,6,7,8]. Comprehensive studies have been conducted to investigate the effects of cooling processing parameters on the quality of sausages [5,6,9,10,11], cooked meat [2,12], and ham [7]. However, there is a lack of studies on the evaluation of different post-storage conditions on the moisture of IVC-cooled sausages using hyperspectral imaging.

Hyperspectral imaging is a powerful non-destructive analytical technique that combines imaging and spectroscopy to capture detailed spectral information from an object across a wide range of wavelengths. In comparison to traditional imaging methods, which capture only a few discrete bands of light (e.g., red, green, blue), hyperspectral imaging collects data at hundreds or even thousands of contiguous narrow spectral bands, typically covering the ultraviolet, visible, and near-infrared regions of the electromagnetic spectrum. It allows the visualization of measured reference parameters across various spots within samples [13,14,15,16] and provides detailed spatial and spectral information, enabling a wide range of applications in research, industry, and environmental monitoring [14]. When coupled with chemometrics analysis, it has proven to be a robust and emerging non-destructive technique that is extensively utilized in the analysis of pork [17,18,19], lamb [20], beef [21,22], chicken [23], ham [24], and processed meat [13,16]. Wang and He (2019) successfully classified Cantonese sausage quality non-invasively using hyperspectral imaging, achieving a predictive accuracy of 100% [25]. An assessment of the physicochemical and microbial attributes of bratwurst, packaged in a 60 μm polyvinylidene chloride coated with polyamide (PVDC/PA) film after 20 days of storage at 4 °C, was conducted [26]. It can thus be concluded that hyperspectral imaging is an emerging method with widespread applicability to various types of meat products. 

Citrus fruits, which are extensively cultivated globally for commercial purposes, serve as a crucial source for the fruit juice, jams, and jellies industry, contributing significantly to job creation and economic benefits [27,28]. The citrus-processing sector reportedly produces over forty million tons of waste annually [29]. Considering that orange production in Shizuoka prefecture accounts for only about 11% of Japan’s total, it indicates a potential tenfold increase in waste production [29]. However, managing this waste whilst adhering to Japan’s Food Recycling Law entails significant costs. Typically, waste orange peels are either directly discarded in landfills as fertilizer, used as animal feed, or sold as dried orange peels to China for integration into Chinese herbal medicine. From an economical point of view, recycling waste orange peels can address environmental concerns while serving as a valuable resource for extracting flavonoids for the pharmaceutical industry [30]. Hesperidin, a flavonoid compound that is predominantly present in orange peels, possesses antioxidative, anti-inflammatory, and anti-cancer properties [31,32], exhibiting potential in combating severe acute respiratory syndrome coronavirus 2 (SARS-CoV-2) [33]. Hesperidin and rutin possess a stronger binding affinity to the main protease of coronavirus disease 2019 (COVID-19) compared to nelfinavir [33,34], indicating their potential as promising candidates for COVID-19 therapeutics.

Sausages, which are made from minced meats, have been a beloved culinary choice for millennia. Although the precise inception of the sausage remains elusive, these flavorful delicacies have historically been savored during yearly celebrations and ceremonial events. Natural casings are favored for use in various sausage manufacturing processes because of their unique tenderness and satisfying texture. In order to minimize the burst incidence of the natural casing without compromising its special tenderness, natural casings were modified by different concentrations of surfactant solution and stored in slush salt with varying additions of lactic acid [35]. The modified casing became more porous, and this allowed the pressure generated from the stuffing, cooking, or immersion vacuum cooling to be released via this porous structure, which led to a reduction in the burst incidence. However, moisture can be influenced if the water can easily “escape” from this porous structure. Moisture stands out as a critical chemical parameter in agricultural products. In meat, water constitutes over 70% of muscle composition, highlighting its pivotal role in preservation methods like curing, smoking, and drying. Controlling moisture becomes essential for maintaining sausage quality and safety, as it interacts with lipids, influencing critical attributes like flavor, juiciness, texture, and appearance [36]. Furthermore, moisture significantly impacts microbial growth, thereby directly influencing the sausage’s shelf life. Previous studies have evaluated the moisture of minced pork [37,38], beef slices at different stages of dehydration [39], the moisture content in salmon fillets [40], and moisture loss in meat analogues [41]. However, little information has been published on the moisture content of immersion vacuum-cooled sausages stuffed in the modified casing with orange extracts. This study is thus to apply hyperspectral imaging to detect the moisture content of sausage after immersion vacuum cooling under different storage conditions.

## 2. Materials and Methods

### 2.1. Sausage Preparation

Sausage batter was made using small pieces of lean pork (70%) and back fat (30%), which were then thoroughly mixed with spices and Chinese white wine (52% ethanol content, *v*/*v*). Natural casings obtained from a local company (Pakumogu.com, Niigata Prefecture, Japan) were desalted before being immersed in a surfactant solution containing soy lecithin and soy oil. Based on previous findings regarding optimal textural properties, antioxidant activities (in vitro) [29], and pH performance [42], the concentrations of soy lecithin (SL) and soy oil (SO) were determined to be 1.11% (g/mL) and 0.85% (g/mL), respectively. The lactic acid concentration in solid salt was 22.5 mL/kg, with a residence time of 66 min. The surfactant solutions (SL and SO) and orange extracts (0.55%, g/mL) were prepared using distilled water, stirred at 325 rpm with magnetic agitation, and heated to 60 °C. After cooling to 25 °C, two groups of casings were immersed in the surfactant solution with orange extracts (SSOE) and without orange extracts (SS, as a control). Subsequently, the casings were removed without rinsing and stored in slush salt containing lactic acid for the designated residence time. Before sausage stuffing, the modified casings underwent a 10 min rinsing process to remove any residual modified solution and slush salt using distilled water. The orange extracts were obtained from ultrasound-assisted extraction of waste from Valencia sweet oranges (*Citrus sinensis*). The details of the extraction process can be found in the study by Feng (2022) [30]. A stuffing machine was used to fill the sausage batter into modified casings treated by SSOE and SS, followed by sectioning via twisting.

### 2.2. Sausage Smoking and Immersion Vacuum Cooling Procedures

Raw sausages were hung in a digital electric smoker chamber (MB20071117, MasterBuilt, Columbus, GA, USA). The smoker machine was preheated for 2 h before the experiments were performed, and the temperature was maintained at 135 °C. The wood sawdust was oak and smoking time was maintained at 135 °C for 1 h. A K-type thermocouple located in the vacuum chamber, which was inserted perpendicularly from the surface to the core, was employed to measure the core temperatures of the sausages. After smoking, the core temperature of the sausages was 63 ± 1 °C. Nine sections of sausages were sterilely cut after smoking and immediately transferred to a 1000 mL glass beaker. Distilled water at 20 °C was added into the beaker to fully immerse the sausages, and the water level was maintained 1 cm above the sausage surface. To prevent water splashing, the beaker was sealed with cling film featuring six perforated holes to allow vapor to escape. Seven beakers were concurrently placed in a vacuum chamber (JS-20QE; Miura Co. Ltd., Ehime, Japan). The vacuum system possesses a pressure transducer to observe the chamber pressure during the cooling process. It took 12 ± 1 min for the sausages to cool down to 4 °C.

The IVC chamber and beakers were sterilized with 75% alcohol. After cooling, each section of the sausage was promptly vacuum-sealed (90% vacuum level) using a low-density polyethylene vacuum bag, which had been sterilized with UV light and measured 15 × 20 cm. Both the control casing sausages and the modified casing sausages were randomly divided into three groups and stored in dark, hygienic incubators with approximately three different conditions: 

Group A: samples were stored at 4 °C for 41 days; 

Group B1 temperature transfer cycle: samples were stored at 4 °C, subjected to 30 °C on the 5^th^ day for 24 h, and then the temperature was reduced to 4 °C again until further analysis (4 ➝ 30 (24 h) ➝ 4);

Group B2 temperature transfer cycles: samples were stored at 4 °C, subjected to 30 °C on the 5^th^ day for 24 h, reduced to a temperature of 4 °C, and then subjected to 30 °C on the 15^th^ day for 36 h and finally reduced to 4 °C until further analysis (4 ➝ 30 (24 h) ➝ 4 ➝ 30 (36 h) ➝ 4);

Group B3 temperature transfer cycles: samples were stored at 4 °C, subjected to 30 °C on the 5^th^ day for 24 h, reduced to 4 °C, subjected to 30 °C on the 15^th^ day for 36 h and reduced to 4 °C, and then subjected to 30 °C on the 25^th^ day for 48 h and finally reduced to 4 °C until further analysis (4 ➝ 30 (24 h) ➝ 4 ➝ 30 (36 h) ➝ 4 ➝ 30 (48 h) ➝ 4)

Group C: samples were stored at 30 °C for 41 days.

### 2.3. Extraction and Processing of Hyperspectral Data

The sausage sample, including both its front and back sides, was aligned perpendicular to the hyperspectral camera to optimize its imaging within the measuring range. The imaging process utilized a hyperspectral camera (model NH-4-KIT, EBA Japan, Tokyo, Japan) employing a push-broom line-scanning technique, capturing 151 contiguous spectra with an exposure time of 12.47 ms. To ensure uniform illumination and prevent shadows, a white sheet was utilized, and three halogen lamp lights were strategically positioned around it. To create a distinct contrast, a black sheet was employed as a black background with ice bags placed under it to maintain the temperature. Two fans were positioned beside the halogen lamps to mitigate any significant temperature increase caused by their heat. Imaging calibration was conducted using the following equation:

(1) $$R_{\mathrm{calibrated}\;\mathrm{image}}=\frac{R_{\mathrm{original}}-R_{\mathrm{dark}}}{R_{\mathrm{white}}-R_{\mathrm{dark}}}$$

The original reflectance image is denoted as R_original_, while R_white_ and R_dark_ represent the reflectance images of white (obtained from a 100% white reference) and dark (captured by covering the camera lens), respectively. A segmentation criterion based on thresholding was applied to identify the sausage samples in the images. Consequently, an initial segmentation mask was generated, selecting all pixels with a reflectance at 695 nm greater than 0.075. Additionally, to increase the number of samples for model creation, the mean spectrum of each sausage was not directly measured; instead, the images were divided into 15 segments, representing cross-sections of the same area. From each segment, the mean reflectance spectrum was extracted. Absorbance spectra were calculated from reflectance through the formula

(2) $$\mathrm{Absorbance}=-\log_{10}R$$

All these digital image processes were conducted using MATLAB (R2023b; MathWorks Inc., Natick, MA, USA). Hyperspectral images were taken on days 10, 19, and 41, with four sections from sausages stuffed with modified casings treated by SSOE and SS, respectively.

### 2.4. Moisture Content Measurement

The minced sausage fillings (5 g), after hyperspectral imaging procedures, were put in an oven set to a temperature of 105 °C for 24 h until a constant weight was reached. The moisture content was calculated as shown below:

(3) $$\mathrm{Moisture}\;\mathrm{content}\;(\%)=\frac{W_{1}-W_{2}}{W_{1}}\;\times \;100\%$$

where W_1_ and W_2_ were the weights before and after drying. The moisture content measurements were conducted on days 10, 19, and 41 in triplicates. 

### 2.5. Establishment and Evaluation of the Regression Model

Several pre-processing techniques, including normalization, standard normal variate (SNV), multiplicative scatter correction (MSC), first derivative (1^st^ Derivative), and a combination of SNV + 1^st^ Derivative, were applied to both reflectance and absorbance spectra before the multivariate analysis was conducted.

A partial least squares regression (PLSR) model was developed by utilizing regression coefficients and weight values associated with specific wavelengths.

(4) $$\mathrm{PLSR}=\sum_{i=1}^{n}W_{i}R_{i}+A$$

where W_i_ represents the i^th^ wavelength, R_i_ stands for the corresponding regression coefficient originating from the PLSR model, and A is the constant of the PLSR model. 

In this study, PLSR models were utilized to establish the relationship between the spectra of the samples and their respective moisture content values. PLSR is a statistical method used to model the relationship between a set of independent variables (spectra in this study) and a dependent variable (moisture in this study). It is particularly useful when there are many independent variables, when there is multicollinearity among them, or when there are more independent variables than observations [32]. In PLSR, the main goal is to find a set of latent variables that capture the maximum covariance between the independent variables and dependent variables. These latent variables are linear combinations of the original variables [16].

Sample selection was performed using the Kennard–Stone algorithm [43]; two-thirds of the dataset were allocated for calibration and one-third for prediction. This algorithm, widely used in chemometrics, selects representative samples by iteratively choosing the points with the maximum distance between them. It considers factors like variability and distribution, ensuring a diverse and informative sample set for analysis. In order to avoid overfitting phenomena, the separation in these sets was of complete sausages. This means that the fifteen segments of each piece were assigned to a unique set.

Model accuracy was evaluated using metrics such as the determination coefficient in calibration (R_c_^2^), prediction (R_p_^2^), and full cross-validation (R_cv_^2^), as well as the root mean square error of calibration (RMSEC), prediction (RMSEP), and full cross-validation (RMSECV), alongside the absence of a significant lack of fit. Typically, a robust PLSR model demonstrates a high R^2^ coupled with a low RMSE, alongside minimal discrepancies between RMSEC and RMSECV [5]. Cross-validation was used as an internal validation methodology to evaluate the residual of each sample. This allowed, on the one hand, to identify the possible presence of spectral outliers and also to evaluate the suitability of the calibration and prediction sets chosen by the Kennard–Stone method. In addition, this cross-validation also made it possible to estimate the number of latent variables of the calibration. Model development and goodness-of-fit calculations were performed using the Statistics and Machine Learning Toolbox within MATLAB (R2023b; MathWorks Inc., Natick, MA, USA).

### 2.6. Prediction Map Development

The moisture content prediction maps of sausages employing various modified casings were generated utilizing calibration models that integrate the entire spectrum. Due to the ability of hyperspectral imaging to capture comprehensive spatial and spectral details within a 3D matrix, a transformation to a 2D matrix is necessary. This process, which is called unfolding, converts a three-dimensional matrix, such as a hypercube, into a flat matrix where each row is a pixel and each column is a wavelength. In this case, it is necessary to add two new variables indicating the spatial coordinates of the pixel in question. Multiplying this matrix by its associated regression coefficient facilitates restructuring the vector, thereby producing a 2D color image. This method effectively illustrates the color discrepancies in sausages utilizing a linear color scale, thereby presenting a distribution map of color variations. All computations were executed utilizing MATLAB software (R2023b; MathWorks Inc., Natick, MA, USA).

### 2.7. Statistical Analysis for Multifactor

The differences among different casing treatments, storage days, and storage conditions influenced the moisture content during 41 days of storage by General Linear Model (GLM) from SPSS software (Statistics 28, IBM, Armonk, NY, USA). The aforementioned multifactor analysis of variances was initially examined to determine if there were any interaction effects among the variables. If the interactive effects of those independent variables are significant at a 5% significance level, simple effect analysis will be used for further analysis. If the interactive effects are insignificant, a multifactor analysis of variance will be employed for the post-analysis.

## 3. Results and Discussion

### 3.1. Effects of Independent Variables on Sausage Moisture Content

The GLM results show that interaction effects were not significant (*p* > 0.05), indicating that the interactions of different casing treatments, storage days, and storage conditions had no effects on moisture content. Although the moisture content was not significantly influenced by the three independent variables mentioned above (*p* > 0.05), the samples stuffed in modified casing treated with orange extracts (59.05 ± 5.87%) and the control (52.02 ± 7.10%) showed the highest and lowest moisture content, respectively, after 41 days of storage at 4 °C (Table 1). It seems that adding orange extracts can compensate for the loss of moisture content during IVC processing and the porous modified casing structure during long-term storage at lower temperatures. Orange extracts may contain compounds that have hydrophilic properties, indicating they have an affinity for water. When incorporated into the sausage formulation, these compounds may attract and retain moisture within the sausage matrix, leading to higher moisture content compared to sausages without the extract. Moreover, certain components present in orange extracts, such as sugars or polyols, may act as humectants. Humectants are substances that can absorb moisture from the surrounding environment and retain it within the product. Therefore, the presence of orange extracts in the sausage formulation could contribute to higher moisture retention, resulting in increased moisture. In addition, orange extracts may interact with proteins and other components in the sausage matrix, altering their water-holding capacity. This can affect the ability of the sausage to retain moisture during storage, leading to differences in moisture content between sausages with and without orange extracts. It was found that moisture content increased to 48–50% when a higher concentration of soy oil (>2.3%) was combined with an addition of orange extracts between 0.05% and 0.40% [37]. Haque et al. (2020) stated the effects of different additions of orange peel extract on the moisture content of beef muscle during frozen storage [44]. The authors attributed the reduction in cooking loss to enhanced emulsion stability and the high capacity of orange peel extract to retain moisture and fat within the meat matrix [44]. 

Regarding the effects of multiple temperature fluctuation cycles on moisture content, differences were observed between sausages with casing that included orange extracts and those without orange extracts in their casings on the 10^th^ day (SSOE vs. SS = 53.54 ± 4.90% vs. 57.04 ± 4.12%) and the 19^th^ day (SSOE vs. SS = 52.80 ± 5.73% vs. 57.49 ± 5.89%). However, by the 41^st^ day, the difference lessened (SSOE vs. SS = 56.57 ± 0.72% vs. 57.58 ± 3.38%). Several potential factors may contribute to the observed phenomena. Temperature fluctuations can have significant effects on moisture content in sausages. It is well known that high temperatures (i.e., 30 °C in the current study) can lead to moisture loss through evaporation, while lower temperatures (4 °C) can cause moisture to condense. This process can occur repeatedly during storage, especially if there are fluctuations in the storage conditions. In the initial stages (the 10^th^ and 19^th^ days), differences in moisture content between sausages with and without orange extracts may be more pronounced due to varying rates of moisture loss or retention under different temperature conditions. The addition of orange extracts to the sausage casing may affect moisture retention or loss. Certain compounds in orange extracts may have hygroscopic properties, meaning they can attract and hold moisture. As a result, sausages with casing containing orange extracts may retain more moisture compared to those without orange extracts in their casing, especially during periods of temperature fluctuation. However, the differences in moisture content between sausages with and without orange extracts may lessen over time. This could be due to several factors. The moisture content may reach equilibrium as sausages undergo aging and the effects of temperature fluctuations become less pronounced. Additionally, any initial differences in moisture content that are attributable to the orange extracts may diminish as the extracts become more evenly distributed throughout the sausage casing during storage. This study simulates how perishable foodstuffs (such as sausage) respond to temperature fluctuations during summer in cold chain transport or due to mishandling by operators. Temperature is vital for ensuring food safety, regulatory compliance, cost efficiency, customer satisfaction, and supply chain integrity. By understanding the factors influencing temperature fluctuations and implementing appropriate control measures, businesses can mitigate risks, optimize operations, and enhance overall performance.

### 3.2. Overview of the Spectra

**Figure 1:**
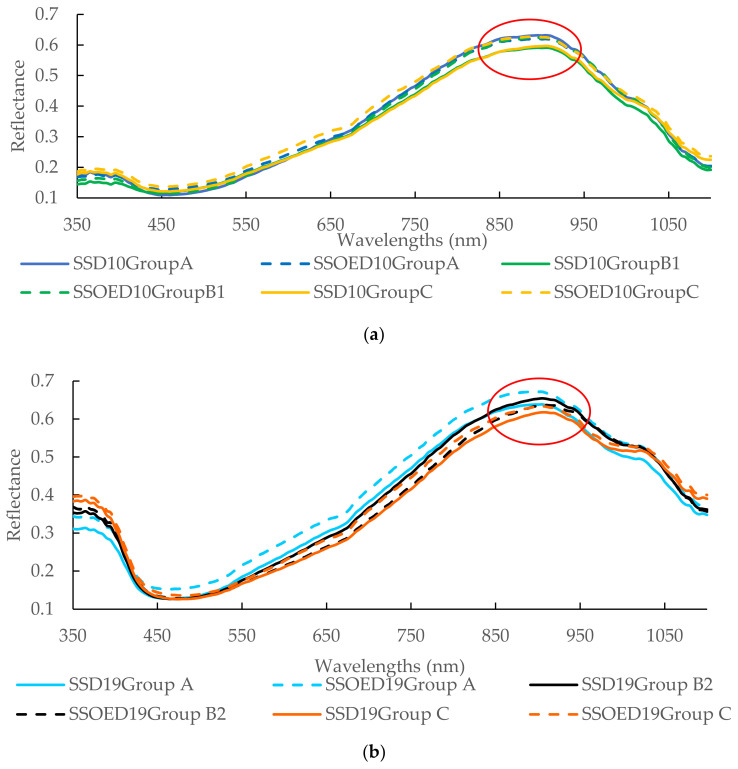

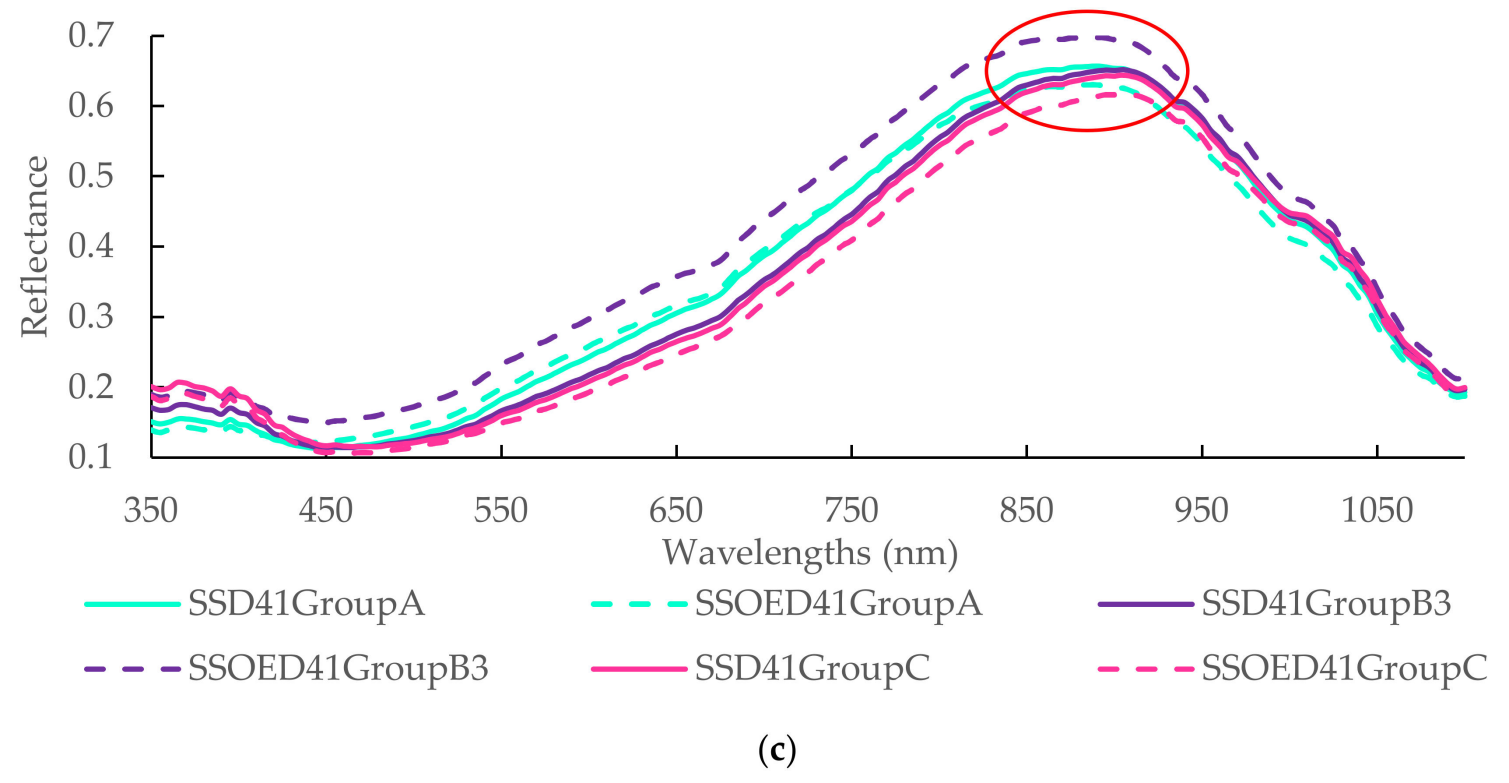
Reflectance of the sausages with different casing treatments and conditions under the 10^th^ (**a**), 19^th^ (**b**), and 41^st^ (**c**) days of storage. Note: SSOE: casing modified by surfactant solution with orange extracts; SS: casing modified by surfactant solution without orange extracts; Group A: samples were stored at 4 °C for 41 days; Group B1 temperature transfer cycle: samples were stored at 4 °C, subjected to 30 °C on the 5^th^ day for 24 h, and reduced to 4 °C until further analysis (4 ➝ 30 (24 h) ➝ 4); Group B2 temperature transfer cycles: samples were stored at 4 °C, subjected to 30 °C on the 5^th^ day for 24 h, reduced to 4 °C, and then subjected to 30 °C on the 15^th^ day for 36 h and finally reduced to 4 °C until further analysis (4 ➝ 30 (24 h) ➝ 4 ➝ 30 (36 h) ➝ 4); Group B3 temperature transfer cycles: samples were stored at 4 °C, subjected to 30 °C on the 5^th^ day for 24 h, reduced to 4 °C, subjected to 30 °C on the 15^th^ day for 36 h and reduced to 4 °C, and then subjected to 30 °C on the 25^th^ day for 48 h and finally reduced to 4 °C until further analysis (4 ➝ 30 (24 h) ➝ 4 ➝ 30 (36 h) ➝ 4 ➝ 30 (48 h) ➝ 4); Group C: samples were stored at 30 °C for 41 days.

The reflectance spectra for the sausages with different casing treatments, storage conditions, and days are illustrated in Figure 1. Spectra were observed to be condensate and tight, and did not show too much difference between different treatments (Figure 1a) on the 10^th^ storage days. The spectra difference became larger concerning different treatments (Figure 1b,c). The reflectance of the sample of SSOED41GroupB3 was the highest, whilst the sample of SSOED41GroupC had the lowest reflectance. The reflectance spectra for sausages with different casing treatments, storage conditions, and days are illustrated in Figure 1. The spectra were observed to be condensed and tight, showing minimal differences between different treatments (Figure 1a) on the 10^th^ day of storage. However, the difference in spectra became more pronounced between treatments (Figure 1b,c). The reflectance of the sample from SSOED41GroupB3 was the highest, while the sample from SSOED41GroupC had the lowest reflectance. Spectral data can provide valuable insights into the chemical composition and molecular structure of substances. Within the visible spectrum, the presence of an absorption band at 430 nm is indicative of the Soret absorption band, which is attributed to the respiratory pigment hemoglobin [45]. Similarly, absorption bands at 560 and 595 nm are associated with respiratory pigments such as oxymyoglobin, which play a crucial role in determining the red color of meat [46]. In the near-infrared (NIR) region, absorptions arise from overtones and combinations of vibrations from functional groups like C-H, N-H, and O-H, which are essential structural components of food molecules. Due to the intricate nature of organic samples, absorption bands in the NIR region tend to be broad and overlap across different areas. Only two subtle absorption bands were detected in the NIR region. These were located at 780 and 980 nm, corresponding to water absorption bands representing the third and second overtones of O-H stretching, respectively. It was observed that the differences in the range from 850 nm to 950 nm became larger with each passing day of storage (as indicated by the red cycle in Figure 1), suggesting a change in moisture content.

### 3.3. Chemometric Analysis

**Figure 2:**
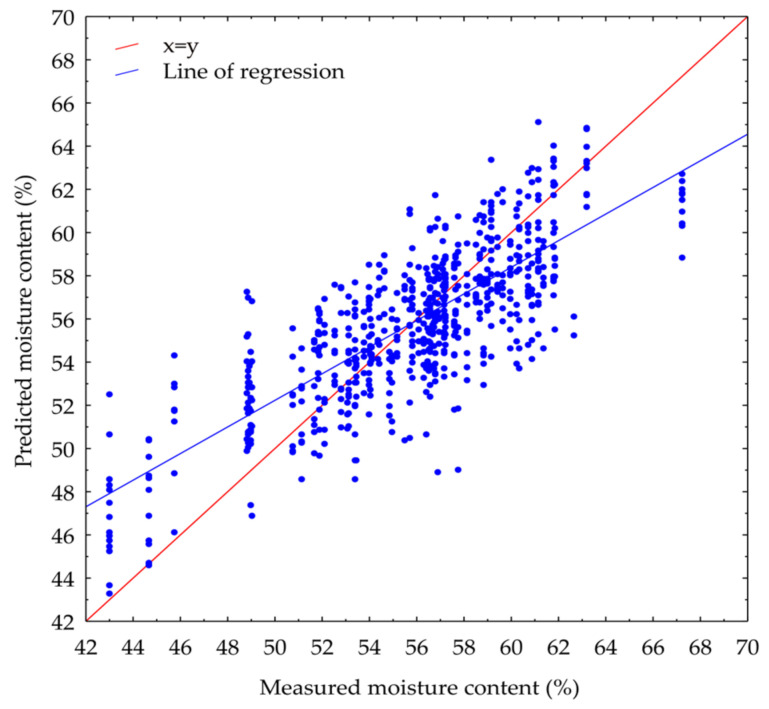
Measured and predicted moisture content in immersion vacuum-cooled sausages with citrus peel extracts for the calibration group using the PLSR model with reflectance spectra pre-treated by standard normal variate.

Chemometrics is the science of applying mathematical and statistical methods to chemical data. It involves using mathematical and statistical techniques to extract useful information from chemical data, especially data obtained from analytical instruments such as spectroscopy, chromatography, and mass spectrometry [47]. It plays a crucial role in areas such as quality control, process optimization, and data interpretation. The statistical parameters of PLSR using different pre-treatments to evaluate moisture content are displayed in Table 2. The SNV pre-processing method improved the R_p_^2^ of reflectance from 0.5358 (raw data) to 0.5683, resulting in a decrease in the RMSEP from 3.0610% to 2.9518%. SNV is reported to eliminate variability in reflectance spectra caused by light scattering [48]. The background noise and baseline drift can be removed by applying the first or second derivatives. Moreover, the subtle spectral features can be accentuated by first and second derivatives [49]. PLSR represents a valuable multivariate data analysis technique for handling datasets with many highly correlated variables in both the independent (X) and dependent (Y) variables. This method has the potential to condense the original predictors into a new set of variables known as latent variables. Widely used due to its ability to mitigate collinearity issues, PLSR also offers flexibility in adjusting the number of variables utilized. The actual measured moisture content and its predicted values from the PLSR model were plotted to visualize the PLSR model performance (Figure 2). It can be observed that the samples are distributed around the regression line, but not too closely, consistent with the R_c_^2^ value of 0.6160. The x = y auxiliary line can be used to judge the performance of a model: if the model’s predicted values (ideally perfectly) match the measured values, all data points should fall on this line. The scatter of data points around the y = x line indicates the model’s variability or spread of errors. If the points are tightly clustered around the line, it indicates low variability, meaning the model’s predictions are consistent. If the points are widely scattered, it suggests high variability, indicating inconsistent predictions. Similarly to the regression line, the data points are widely distributed around the x = y auxiliary line, which may again be attributed to the comparably low R_c_^2^ value. Several factors may affect the prediction accuracy; these factors include sensor noise, atmospheric interference, data pre-processing, sample size, and so on. Although various spectra pre-treatments and other algorithms have been applied, the highest R square of calibration achieved is only 0.6160. Girolamo et al. (2009, 2014) have stated that an *R*^2^ value between 0.66 and 0.81 implies approximate calibration, while an *R*^2^ value between 0.50 and 0.64 is regarded as a usable rough prediction [50,51]. For model evaluation, root mean square error (RMSE) is also very important. It is known that RMSE is a measure of the differences between values predicted by a model or estimator and the actual observed values. It is calculated as the square root of the mean of the squared differences between the predicted and actual values. A lower RMSE indicates that the model’s predictions are closer to the actual observed values, suggesting a better fit of the model to the data. Conversely, a higher RMSE indicates larger prediction errors. As the RMSEP in our study is less than 3%, this could also indicate that the model may be appropriate for some industries.

The fresh and frozen–thawed beef was classified by a hyperspectral imaging sensor associated with machine learning [52]. The authors mentioned that the model’s performance could significantly differ depending on the pre-processing method and the choice of kernel function. The fresh, frozen–stored, and frozen–thawed beef cuts were again discriminated against by hyperspectral imaging. The findings indicated that classification models utilizing feature variables derived from distinct tissue spectra attained superior performances, with accuracy rates of 92.75% for partial least squares discriminant analysis, 97.83% for support vector machine, and 95.03% for backward propagation–artificial neural network. Additionally, a visualization map was introduced to offer comprehensive insights into the alterations in beef cuts’ freshness following freeze–thaw cycles [22]. 

### 3.4. Prediction Map from Representative Sausage

**Figure 3:**
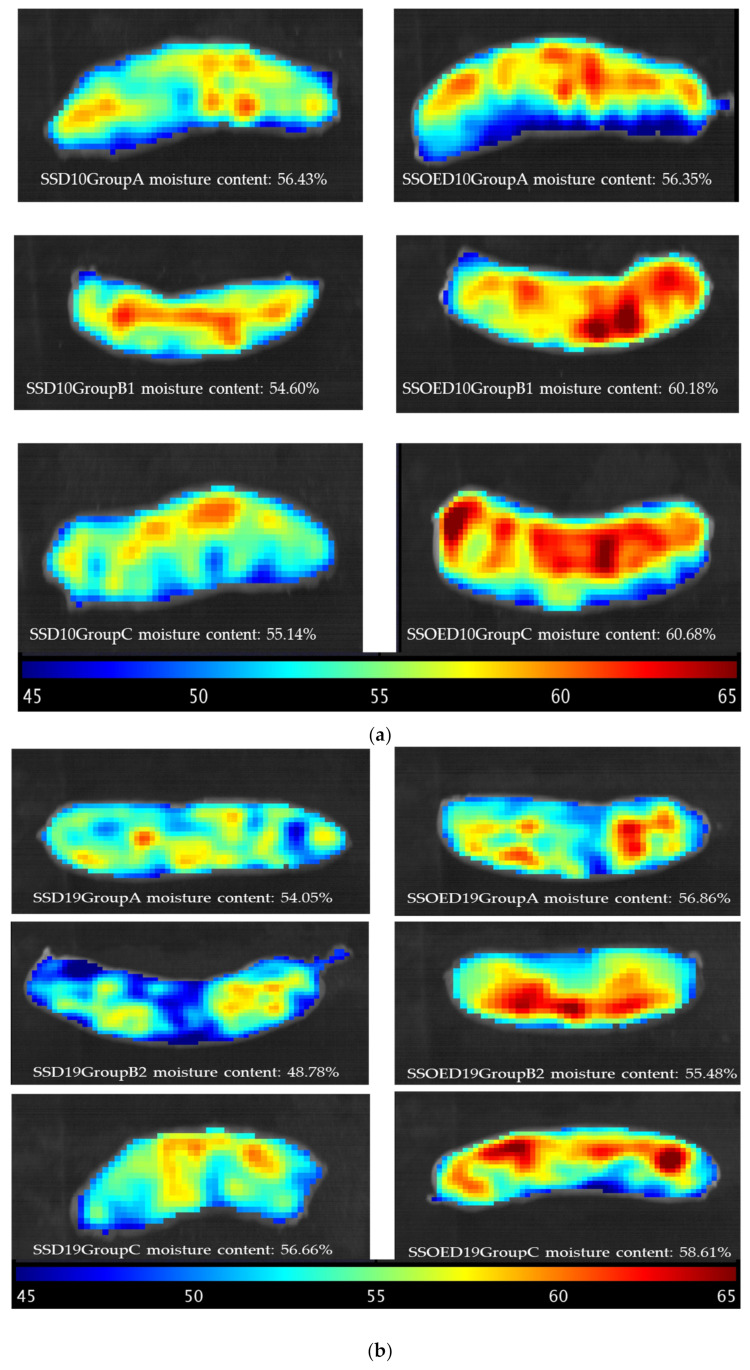

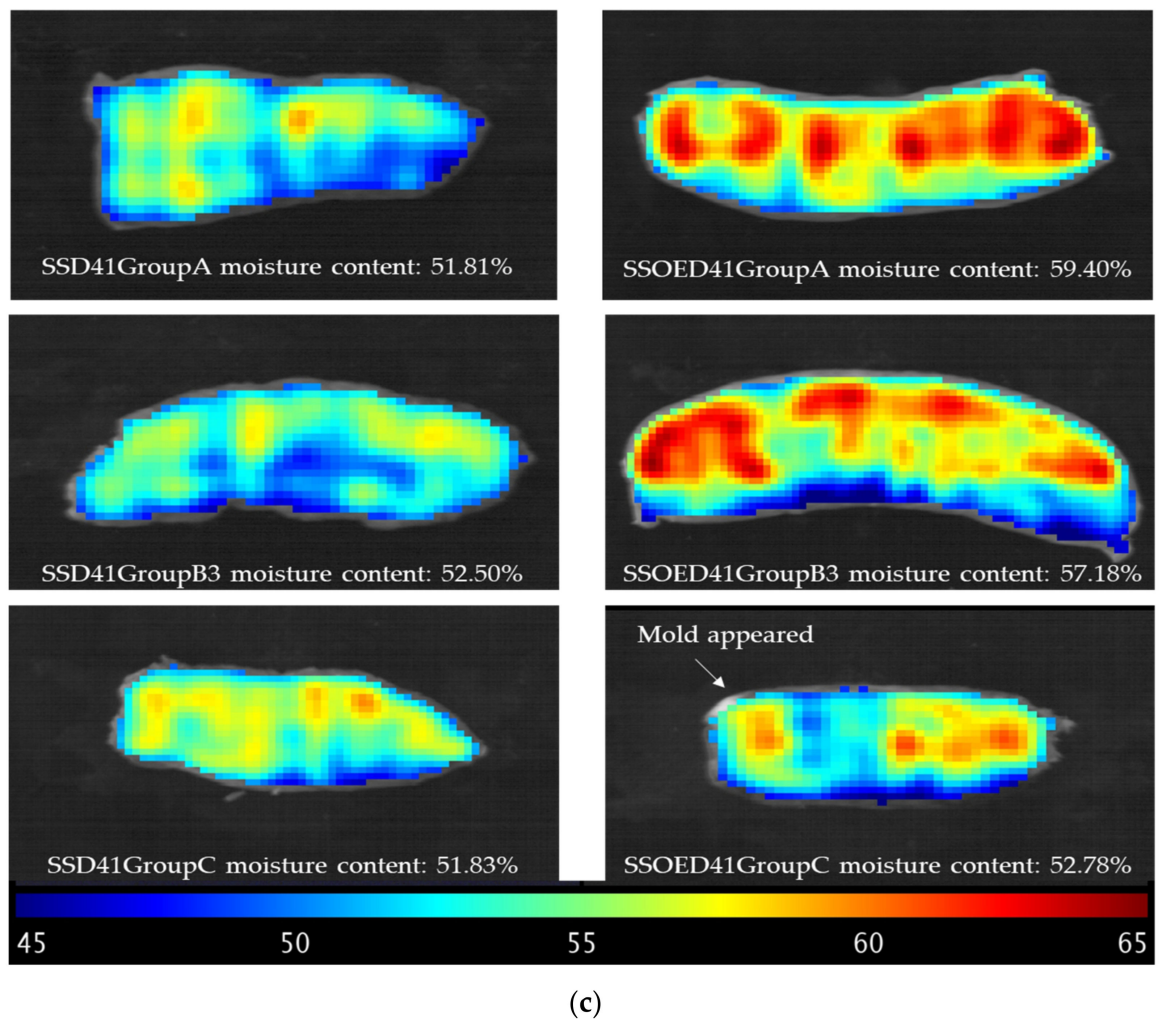
Moisture content prediction map for representative sausages with different casing treatments and conditions on the 10^th^ (**a**), 19^th^ (**b**), and 41^st^ (**c**) days of storage. Note: SSOE: casing modified by surfactant solution with orange extracts; SS: casing modified by surfactant solution without orange extracts; Group A: samples were stored at 4 °C for 41 days; Group B1 temperature transfer cycle: samples were stored at 4 °C, subjected to 30 °C on the 5^th^ day for 24 h, and reduced to 4 °C until further analysis (4 ➝ 30 (24 h) ➝ 4); Group B2 temperature transfer cycles: samples were stored at 4 °C, subjected to 30 °C on the 5^th^ day for 24 h, reduced to 4 °C, and then subjected to 30 °C on the 15^th^ day for 36 h and finally reduced to 4 °C until further analysis (4 ➝ 30 (24 h) ➝ 4 ➝ 30 (36 h) ➝ 4); Group B3 temperature transfer cycles: samples were stored at 4 °C, subjected to 30 °C on the 5^th^ day for 24 h, reduced to 4 °C, subjected to 30 °C on the 15^th^ day for 36 h and reduced to 4 °C, and then subjected to 30 °C on the 25^th^ day for 48 h and finally reduced to 4 °C until further analysis (4 ➝ 30 (24 h) ➝ 4 ➝ 30 (36 h) ➝ 4 ➝ 30 (48 h) ➝ 4); Group C: samples were stored at 30 °C for 41 days.

Hyperspectral imaging provides a significant advantage by offering both spectral information and spatial distribution across samples. Consequently, when regression models are constructed, it becomes feasible to generate distribution maps that enhance the interpretation of results. To achieve this, the pixels within the region of interest were identified and organized into a table, with each pixel’s coordinates being stored for subsequent reconstruction. Similar spectral pre-processing methods used in model creation were applied to these spectra, which were then subjected to matrix multiplication with the regression vector, incorporating the independent term. The resulting values corresponded to moisture content, which was expressed in the same units used during model creation (%). Subsequently, the data were reassembled according to the original image, employing a color scale for improved visualization. These distribution maps were produced alongside the reconstructed RGB image to facilitate understanding. Figure 3 shows the distinct visibility of average moisture content and their locations within the sausage. This facilitates a swift evaluation of the moisture content of representative sausages subjected to different treatments, underscoring the primary advantage of HSI technology over traditional spectroscopic methods. The rationale behind this lies in the fact that pixels with similar spectral characteristics yield comparable predicted values, resulting in consistent representations on the prediction map. Consequently, the varying moisture of sausages with different treatments can be non-invasively detected using hyperspectral imaging. Compared to the traditional method of moisture content measurement, which is tedious and can only obtain the average moisture content, a moisture prediction map can illustrate the concentration and distribution within the sample, aiding in real-time inspection and early mold detection.

Although a comparatively high moisture content may render sausages juicier and more tender, it may also increase the risk of mold growth. As can be seen in Figure 3c, mold grew after 41 days of storage at 30 °C (SSOED41GroupC). This may be due to broken casing caused by cutting, incomplete vacuum seal conditions, as well as a high storage temperature. It is well known that mold requires moisture to grow, so sausages with higher water content are more susceptible to mold growth. Typically, sausages with a water activity (aw) level above 0.85 are at risk of mold growth. Moreover, most molds require oxygen to grow, so aerobic conditions promote mold growth. Sausages whose surfaces are exposed to air are more prone to mold growth than those that are tightly packaged or vacuum-sealed. In the current study, the sausages were vacuum-sealed with a 90% vacuum level. Mold requires organic matter for growth, and sausages, being organic products, provide suitable nutrients for mold growth. Mold growth on sausages typically occurs within a range of 15 °C to 25 °C, with optimal growth typically occurring around room temperature. However, molds can also grow at higher temperatures, albeit more slowly. The growth conditions of *P. nordicum* and *P. verrucosum* in dry-cured sausages were studied by Rodriguez et al. (2015) [53], it was found that the optimum growth for *P. nordicum* took place at 0.94 water activity and 20 °C, whilst for *P. verrucosum*, it occurred at 0.94 and 25 °C. However, these types of mold also grew at 30 °C, although their growth slower than at 20–25 °C at all water activity levels. Accordingly, it can be observed from the prediction map (Figure 3c) that there were areas with high water content (orange range) near the mold growth area.

To prevent mold growth, it is essential to control these factors. This includes proper storage conditions (such as refrigeration or vacuum packaging) to control temperature and moisture, as well as ensuring good sanitation practices to minimize mold contamination. The prediction maps showed exact parts with high water content, which will help to monitor and prevent mold growth.

## 4. Conclusions

The moisture content of immersion vacuum-cooled sausages stuffed in different casing treatments associated with different storage conditions was evaluated by utilizing hyperspectral imaging coupled with chemometrics. Different pre-treatment combinations were applied to raw reflectance and absorbance spectra, revealing that the standard normal variate (SNV) reflectance spectra demonstrated superior performance compared to other pre-processed spectra. The partial least squares regression model, developed using spectra pre-treated with SNV, produced comparable satisfactory results with an R_c_^2^ of 0.6160 and an RMSEP of 2.8130%. The novelties of this study are as follows:

(1) Investigating the moisture contents of immersion vacuum-cooled sausages stuffed in casings modified by various treatments, along with the addition of orange extracts, during fluctuant temperature changes under long-term storage, which has not been evaluated previously. Prediction maps vividly depict the moisture content variations at each pixel in response to different casing treatments. These findings contribute to improving our understanding of the interaction between moisture content and orange extracts, providing valuable insights for future research.
(2) Establishing, for the first time, the relationship between moisture content and spectra obtained from the surface of cylindrical immersion vacuum-cooled sausages with modified casings using PLSR.
(3) The prediction map may play a crucial role in the early detection of mold appearance.

Hyperspectral imaging emerges as a promising tool for the rapid and non-destructive measurement of moisture content, facilitating both quantification and visualization of moisture evolution in sausages during long-term storage and a tool for early mold detection.

## Figures and Tables

**Table 1:** Regression coefficients and analysis of variance for the ATP regression models.

Moisture Content Determined Day	Casing Treatment	Storage Condition	Average Moisture Content (%)
10	SSOE	Group A	56.23 ± 1.30 ^ns^
	SS	Group A	56.78 ± 2.16 ^ns^
	SSOE	Group B1	53.54 ± 4.90 ^ns^
	SS	Group B1	57.04 ± 4.12 ^ns^
	SSOE	Group C	52.53 ± 6.61 ^ns^
	SS	Group C	55.12 ± 2.93 ^ns^
19	SSOE	Group A	55.44 ± 4.56 ^ns^
	SS	Group A	56.95 ± 4.92 ^ns^
	SSOE	Group B2	52.80 ± 5.73 ^ns^
	SS	Group B2	57.49 ± 5.89 ^ns^
	SSOE	Group C	55.74 ± 5.66 ^ns^
	SS	Group C	58.29 ± 2.38 ^ns^
41	SSOE	Group A	59.05 ± 5.87 ^ns^
	SS	Group A	52.02 ± 7.10 ^ns^
	SSOE	Group B3	56.57 ± 0.72 ^ns^
	SS	Group B3	57.58 ± 3.38 ^ns^
	SSOE	Group C	53.70 ± 3.59 ^ns^
	SS	Group C	55.20 ± 3.26 ^ns^

Note: SSOE: casing modified by surfactant solution with orange extracts; SS: casing modified by surfactant solution without orange extracts; Group A: samples were stored at 4 °C for 41 days; Group B1 temperature transfer cycle: samples were stored at 4 °C, subjected to 30 °C on the 5^th^ day for 24 h, and reduced to 4 °C until further analysis (4 ➝ 30 (24 h) ➝ 4); Group B2 temperature transfer cycles: samples were stored at 4 °C, subjected to 30 °C on the 5^th^ day for 24 h, reduced to 4 °C, and then subjected to 30 °C on the 15^th^ day for 36 h and finally reduced to 4 °C until further analysis (4 ➝ 30 (24 h) ➝ 4 ➝ 30 (36 h) ➝ 4); Group B3 temperature transfer cycles: samples were stored at 4 °C, subjected to 30 °C on the 5^th^ day for 24 h, reduced to 4 °C, subjected to 30 °C on the 15th day for 36 h and reduced to 4 °C, and then subjected to 30 °C on the 25^th^ day for 48 h and finally reduced to 4 °C until further analysis (4 ➝ 30 (24 h) ➝ 4 ➝ 30 (36 h) ➝ 4 ➝ 30 (48 h) ➝ 4); Group C: samples were stored at 30 °C for 41 days; ^ns^: not significant.

**Table 2:** Statistical parameters for PLSR using both raw and pre-processed spectra.

Spectra	Treatments	Calibration Group (*n* = 682)		Prediction Group (*n* = 341)		Cross-Validation	
		R_c_^2^	RMSEC (%)	R_p_^2^	RMSEP (%)	R_cv_^2^	RMSECV (%)
Reflectance	Raw data (untreated)	0.5906	2.9030	0.5358	3.0610	0.3724	3.5991
	1^st^ Derivative	0.5766	2.9523	0.5233	3.1018	0.4641	3.3270
	SNV	0.6160	2.8130	0.5683	2.9518	0.5151	3.1518
	SNV + 1^st^ Derivative	0.5879	2.9126	0.5321	3.0730	0.4814	3.2712
	1^st^ Derivative + SNV	0.5863	2.9181	0.5323	3.0725	0.4847	3.2492
Absorbance	Raw data (untreated)	0.5751	2.9574	0.4987	3.1808	0.4667	3.3174
	1^st^ Derivative	0.5939	2.8913	0.5211	3.1088	0.4649	3.3228
	SNV	0.3596	3.6307	0.2984	3.7629	0.4404	3.3961
	SNV + 1^st^ Derivative	0.5923	2.8968	0.5361	3.0600	0.4583	3.3464
	1^st^ Derivative + SNV	0.5920	2.8980	0.5405	3.0453	0.4597	3.3395

Note: SNV: standard normal variate; R_c_^2^: coefficients of determination for calibration; R_p_^2^: coefficients of determination for prediction; R_cv_^2^: coefficients of determination for cross-validation. RMSEC: root mean square error of calibration; RMSEP: root mean square error of prediction; RMSECV: root mean square error of full cross-validation.

## Data Availability

Data are contained within this article.
